# Resident Education During COVID-19 Pandemic: Effectiveness of Virtual Electroencephalogram Learning

**DOI:** 10.7759/cureus.11094

**Published:** 2020-10-22

**Authors:** Sisira Yadala, Krishna Nalleballe, Rohan Sharma, Mitesh Lotia, Nidhi Kapoor, Karthika Durga Veerapaneni, Sukanthi Kovvuru, Sanjeeva Onteddu

**Affiliations:** 1 Neurology, University of Arkansas for Medical Sciences, Little Rock, USA

**Keywords:** resident education, eeg, epilepsy, teaching, medical education, virtual teaching, virtual

## Abstract

Objective

To explore effectiveness of alternative methods of neurology resident electroencephalogram (EEG) learning during COVID-19 pandemic due to social distancing requirements which caused disruption of traditional in-person teaching.

Methods

Virtual EEG learning was instituted using Zoom platform. Residents participated in live, interactive virtual sessions for eight weeks. A pre-test and post-test were administered and a survey was performed at the end of the project.

Results

Based on pre-test and post-test results, there was a significant improvement on average resident test scores. On the survey, 100% agreed (81.8% strongly agreed, 18.2% agreed) that virtual EEG sessions provided a conducive learning environment with easy access while preserving effective communication with the instructor. When compared to traditional EEG reading, 100% agreed (81.8% strongly agreed and 18.2% agreed) that virtual sessions were more accessible, 72.7% agreed (54.5% strongly agreed, 18.2% agreed) that they were more interactive; 81.9% (45.5% strongly agreed, 36.4% agreed) felt more engaged and 90.9% agreed (81.8% strongly agreed, 9.1% agreed) that they were able to attend more sessions. Hundred percent residents (72.7% strongly agreed, 27.3% agreed) felt more confident in their EEG reading and all (81.8% strongly agreed and 18.2% agreed) would sign up for more virtual learning courses.

Conclusions

Virtual EEG education is an efficient method of resident education with improved ease of access while maintaining interactive discussion leading to increased confidence in learners. It should be considered even after resolution of the need for social distancing and its applications in other fields of learning should be further explored.

## Introduction

Traditionally, electroencephalogram (EEG) reading has been taught to neurology residents by epilepsy/neurology attendings in a face to face model. The unprecedented times of COVID-19 pandemic have resulted in a number of challenges for physicians, not only in providing medical care for patients but also created gaps in medical education of residents and fellows. As COVID-19 cases continued to rise, large gatherings and in-person meetings were canceled at our institution in line with the national guidelines for social distancing [1, 2]. Consequently, traditional in-person face to face teaching model of resident education was significantly disrupted and alternative teaching methods needed to be explored.

Though face to face method of instruction is the most common method of EEG teaching, there have been a few studies published regarding the use of pre-recorded training modules or videos with reasonable success [3-5]. To our knowledge, ours is the first report of results from a virtual, live, interactive EEG teaching method for neurology residents.

## Materials and methods

During the COVID-19 pandemic, to mitigate the spread of the virus, persons who came in direct contact with patients with COVID-19 without appropriate personal protective equipment prior to diagnosis needed to be placed in 14-day quarantine. Due to the limited number of neurology house-staff, we used a temporary resident back up model at our institute where 50% of residents were involved in direct patient care in essential services such as ICUs, neurology floor service and neurology consult service while the other 50% were placed on back up at home with a variety of virtual learning opportunities. Residents alternated between the two teams. The backup resident could be called into service if anyone from the essential services team needed to be placed on quarantine. This provided the back-up team residents time to participate in virtual learning.

In an effort to provide education to the residents interested in having an EEG rotation as well as to diversify educational opportunities for residents on the back up team, virtual EEG learning was instituted using Zoom platform. Institutional information technology (IT) security reviewed and approved Health Insurance Portability and Accountability Act (HIPAA) compliance of Zoom. Our institute and Zoom entered into a HIPAA Business Associate Agreement (BAA) specific to protecting the privacy and security of patient health information (PHI).

Residents participated in two live, interactive virtual EEG reading sessions per day for a total of approximately 3-5 hours each day, five days a week. A Zoom invitation was sent out twice a day and all residents who were available joined the virtual conference. In addition to the residents assigned to the EEG rotation, residents on the back up team, as well as the residents on floor and ICU teams were able to join the virtual classes as and when available.

During the virtual teaching session, the instructor shared their screen with the audience, which enabled the residents to look at the EEG at the same time as the instructor. Participants accessed the sessions via computers or tablets or smartphones based on availability. All participants had a microphone, which enabled interaction with the instructor and in between residents. The video feature, chat box and annotate functions were used occasionally as needed. The annotate function enabled to virtually draw on the screen which was used as a teaching aid when illustrations with hand drawings were required.

The residents were administered a pre-test with 23 questions prior to the virtual EEG learning project and a post-test with 23 questions after completion of the project. The tests were administered using the Blackboard virtual learning environment. Due to COVID social distancing requirements, residents were allowed to take the tests at locations of their convenience. After completion of eight weeks, a survey was given to the residents with questions regarding their experience and opinion regarding virtual EEG learning method.

## Results

There were a total of 11 neurology residents across PGY2 to PGY4 years at our institute at the time of this project, out of which, approximately 5-9 residents were able to join each session. This virtual EEG teaching project lasted for eight weeks.

**Figure 1:**
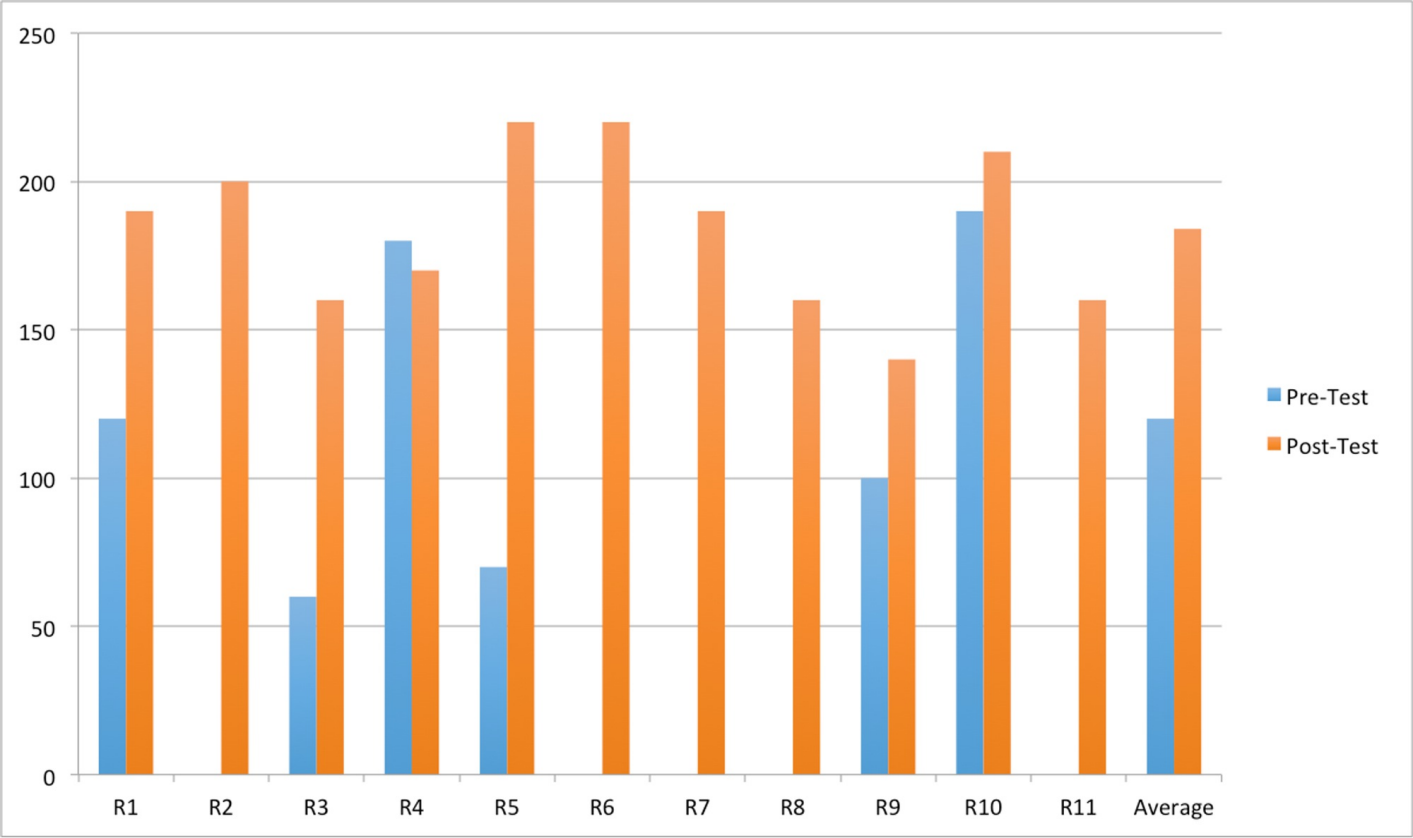
Pre-test and post-test score comparison R-Resident, Residents with absent blue bars did not take pre-test.

Six residents took the pre-test, of which two residents were in PGY-2, two residents were in PGY-3 and two residents were in PGY-4. Out of a maximum possible score of 230, the average score of the residents on the pre-test was 120 (Table 1, Figure 1). All 11 residents took the post-test and the average score on the post-test was 184 out of a maximum possible score of 230. Amongst the residents who took both pre-test and post-test, the average pre-test score was 120 and average post-test score was 182.

**Table 1:** Pre-test and post-test scores R – Resident. Maximum possible test score was 230. NA – Not Applicable (Resident did not take pretest)

	Pre-test Score	Post-test Score
R1	120	190
R2	NA	200
R3	60	160
R4	180	170
R5	70	220
R6	NA	220
R7	NA	190
R8	NA	160
R9	100	140
R10	190	210
R11	NA	160
Average of all PGYs	120	184

All 11 residents completed a survey after the completion of the virtual EEG learning project (Tables 2, 3). All residents found limitations in the traditional face-to-face learning with attending that included difficulty coordinating reading times, other clinical duties and non-availability of the EEG reader due to other responsibilities. Based on the survey results, 100% of the residents agreed (81.8% strongly agreed, 18.2% agreed) that the virtual EEG sessions provided a conducive environment for learning, they were able to access the sessions with much ease, they were able to communicate with the instructor when needed and that the instructor was able to encourage attendee participation. Hundred percent of the residents agreed (72.7% strongly agreed, 27.3% agreed) that they felt more confident in their EEG reading skills based on their experience with virtual EEG learning. Hundred percent of the residents agreed (63.6% strongly agreed, 36.4% agreed) that they were satisfied with their overall experience. Hundred percent of the residents agreed (81.8% strongly agreed and 18.2% agreed) that when compared to traditional EEG reading, virtual EEG reading was more accessible. When compared to traditional EEG reading, 72.7% residents agreed (54.5% strongly agreed, 18.2% agreed) that virtual EEG reading was more interactive while 27.3% residents were neutral. When compared to traditional EEG reading, 81.9% residents agreed (45.5% strongly agreed, 36.4% agreed) that virtual EEG reading kept them more engaged while 18.2% residents were neutral. When compared to traditional EEG reading, 90.9% residents agreed (81.8% strongly agreed, 9.1% agreed) that they were able to attend more virtual EEG reading sessions while 9.1% residents were neutral. Hundred percent of the residents strongly agreed that they were able to attend more sessions due to the ease of access and 100% of the residents agreed (81.8 strongly agreed and 18.2% agreed) that they would sign up for more virtual learning courses based on this experience.

**Table 2:** Survey results

	N	Percentage %
Describe the various types of EEG learning sessions prior to this virtual session (May check multiple answers):		
In person sessions with attendings	9	81.8
Chairman's Rounds	5	45.5
Noon Lectures	9	81.8
Self-Study	8	72.7
Group sessions with attending	1	9.1
Prior to the virtual EEG sessions, what were the limitations in learning the EEG modules (May check multiple answers):		
Difficulty coordinating EEG reading times	10	90.9
Other clinical duties	10	90.9
Non-availability of the EEG reader in person	10	90.9
Other - Used of get exhausted in clinic and lost interest in EEG reading	1	9.1
Total number of residents who took the survey was 11		

**Table 3:** Survey results

	Strongly Agree (%)	Agree (%)	Neutral (%)	Disagree (%)	Strongly Disagree (%)
The virtual EEG sessions provided a conducive environment for learning	81.8	18.2	0	0	0
I was able to access the sessions with much ease.	81.8	18.2	0	0	0
I was able to attend more sessions due to the ease of access.	100	0	0	0	0
I was able to communicate with the instructor when needed	81.8	18.2	0	0	0
The instructor was able to encourage attendee participation	81.8	18.2	0	0	0
Based on my experience with the virtual EEG, I feel more confident in my EEG reading skills	72.7	27.3	0	0	0
I am satisfied with my overall experience	63.6	36.4	0	0	0
When compared to traditional EEG reading this virtual EEG learning was more interactive	54.5	18.2	27.3	0	0
When compared to traditional EEG reading this virtual EEG learning was more accessible	81.8	18.2	0	0	0
When compared to traditional EEG reading this virtual EEG learning kept me more engaged	45.5	36.4	18.2	0	0
When compared to traditional EEG reading, I was able to attend more virtual EEG sessions	81.8	9.1	9.1	0	0
I would sign up for more virtual learning courses based on this experience	81.8	18.2	0	0	0

## Discussion

With a sudden change in the dynamics of the operations due to COVID-19, physicians have shown great adaptability in not only providing medical care but also in deploying novel teaching techniques. Based on above results, virtual EEG teaching appears to be a very effective method of educating neurology residents. There was a significant improvement in the post-test results compared to the pre-test results, which provides an objective evidence of the efficacy of this teaching method. The residents gave a strong positive feedback based on the survey results that this method provided a conducive environment for learning, they were able to access the sessions with much ease, they were able to communicate with the instructor when needed, the instructor was able to encourage attendee participation, they felt more confident in their EEG reading skills after the project, and they were satisfied with their overall experience. The residents also felt that when compared to traditional EEG reading, virtual EEG reading was more accessible. A majority of the residents felt that when compared to traditional EEG reading, virtual EEG reading was more interactive, kept them more engaged and they were able to attend more virtual EEG reading sessions, while a minority of residents were neutral on these questions though no one disagreed.

A survey evaluating the neurophysiology knowledge gap showed that amongst graduating PGY-4 residents, confidence in reading EEGs was at a median of 60% and 67% on a scale of 0% to 100% for the level 4 ACGME milestones. Neurology program directors suspected that up to 15% of residents may graduate not meeting the level 4 milestones in EEG. Lack of time was cited as the most common obstacle, which included lack of a formal rotation and inpatient burden. However, residents thought that reading EEGs with an attending was the best way to learn [1]. Early integrated exposure to EEG was proposed as a possible intervention to improve EEG reading proficiency of neurology residency graduates and lack of time was cited as the most common obstacle including lack of sufficient formal EEG teaching and other responsibilities such as inpatient burden similar to findings on our resident survey [6]. Our study demonstrates that virtual EEG teaching method, overcomes both these barriers at least partially by providing easy access to EEG teaching sessions to all residents including PGY-2 residents who are often busy on inpatient services. This is especially important in smaller neurology residency programs with limited number of epilepsy faculty who may be less readily available to residents due to their overwhelming patient care responsibilities.

There were more test takers for the post-test than the pre-test, which we believe is due to increased engagement and interest amongst residents as they realized the efficacy of this teaching method. The residents reporting that virtual EEG learning was more interactive with better engagement than traditional methods appears to be due to the virtual interface providing a great platform for discussion and interactions amongst the residents and understand each other’s perspective without compromising their access to the instructor. In addition, the inpatient and consult residents were able to login during down time such as before rounds, during table rounds, other unexpected breaks, etc. and provide real-time clinical updates which provided a great premise for good discussions. Limitations of this study include the overall low number of participating residents, low number of residents who took the pre-test and participation of only one center.

## Conclusions

Based on our project, virtual EEG teaching appears to be a very efficient method of resident learning and should be strongly encouraged. Though this method was introduced as an alternative to traditional face to face teaching during COVID-19 pandemic due to need for social distancing, it has been found to be so effective that it needs to be considered as a part of regular curriculum even after the pandemic resolves and social distancing is no longer needed. Though this project was primarily done for EEG teaching, in view of the robust positive results, it would very likely be applicable to other fields of learning as well and should be further explored and studied.
